# Genome-wide mutational landscape of mucinous carcinomatosis peritonei of appendiceal origin

**DOI:** 10.1186/gm559

**Published:** 2014-05-29

**Authors:** Hakan Alakus, Michele L Babicky, Pradipta Ghosh, Shawn Yost, Kristen Jepsen, Yang Dai, Angelo Arias, Michael L Samuels, Evangeline S Mose, Richard B Schwab, Michael R Peterson, Andrew M Lowy, Kelly A Frazer, Olivier Harismendy

**Affiliations:** 1 Division of Genome Information Sciences, Department of Pediatrics and Rady Children’s Hospital, University of California San Diego, La Jolla, CA, USA; 2 Division of Surgical Oncology, Department of Surgery, University of California San Diego, La Jolla, CA, USA; 3 Department of Medicine, University of California San Diego, La Jolla, CA, USA; 4 Bioinformatics Graduate Program, University of California San Diego, La Jolla, CA, USA; 5 Moores UCSD Cancer Center, University of California San Diego, La Jolla, CA, USA; 6 Department of Pathology, University of California San Diego, La Jolla, CA, USA; 7 Clinical and Translational Science Institute, University of California San Diego, La Jolla, CA, USA; 8 Institute for Genomic Medicine, University of California San Diego, La Jolla, CA, USA; 9 RainDance Technologies, Lexington, MA, USA; 10 Department of General, Visceral and Cancer Surgery, University of Cologne, Köln, Germany

## Abstract

**Background:**

Mucinous neoplasms of the appendix (MNA) are rare tumors which may progress from benign to malignant disease with an aggressive biological behavior. MNA is often diagnosed after metastasis to the peritoneal surfaces resulting in mucinous carcinomatosis peritonei (MCP). Genetic alterations in MNA are poorly characterized due to its low incidence, the hypo-cellularity of MCPs, and a lack of relevant pre-clinical models. As such, application of targeted therapies to this disease is limited to those developed for colorectal cancer and not based on molecular rationale.

**Methods:**

We sequenced the whole exomes of 10 MCPs of appendiceal origin to identify genome-wide somatic mutations and copy number aberrations and validated significant findings in 19 additional cases.

**Results:**

Our study demonstrates that MNA has a different molecular makeup than colorectal cancer. Most tumors have co-existing oncogenic mutations in *KRAS* (26/29) and *GNAS* (20/29) and are characterized by downstream PKA activation. High-grade tumors are *GNAS* wild-type (5/6), suggesting they do not progress from low-grade tumors. MNAs do share some genetic alterations with colorectal cancer including gain of 1q (5/10), Wnt, and TGFβ pathway alterations. In contrast, mutations in *TP53* (1/10) and *APC* (0/10), common in colorectal cancer, are rare in MNA. Concurrent activation of the *KRAS* and *GNAS* mediated signaling pathways appears to be shared with pancreatic intraductal papillary mucinous neoplasm.

**Conclusions:**

MNA genome-wide mutational analysis reveals genetic alterations distinct from colorectal cancer, in support of its unique pathophysiology and suggests new targeted therapeutic opportunities.

## Background

Mucinous neoplasms of the appendix (MNA), though rare, represent a common subtype of appendiceal tumors with an incidence of one per million in the US [1]. Primary MNAs are classified histologically as either low-grade appendiceal mucinous neoplasm (LAMN) showing no classic infiltrative invasion or mucinous adenocarcinoma [2] which reflects a high-grade, invasive form of the disease associated with a worse prognosis. MNAs are often diagnosed after primary tumor rupture and metastasis to the peritoneal cavity and designated as high or low histological grade. The metastatic tumor cells often secrete large quantities of extracellular mucin resulting in the clinical syndrome known as mucinous carcinomatosis peritonei (MCP) or pseudomyxoma peritonei (PMP). Due to its location in the peritoneal cavity, the organ of origin of MCP has been debated for a long time [3], but a more systematic histology assessment [4] as well as molecular characterization [5] has concluded that the vast majority of MCP originates from the appendix. Once peritoneal metastasis has occurred, disease progression, though relatively indolent, is frequently fatal [2,6].

The primary treatment of low-grade MCP is surgical. Patients with high-grade MCP and those with inoperable disease typically receive therapies approved for colorectal cancer (CRC), although there is a limited understanding of their efficacy. Furthermore, the genome-wide mutational landscape of MNAs remains uncharted and its genetic relationship to CRC has not been thoroughly studied. As a consequence, targeted therapies, which have dramatically improved the outcome in many cancer types, including CRC, have not yet impacted MNA treatment. A few focused studies of MNAs have reported activated *KRAS* in 50% to 100% of the cases [5,7-10], loss of heterozygosity of *APC* in one patient [11,12], and p53 over-expression in 44% of the cases [10]. A recent study identified frequent mutations in *GNAS* in LAMN, which were associated with high mucin production [13]. However, the molecular study of MNA remains technically challenging due to: (1) its very low incidence; (2) the poor quality of available MCP samples which have low cellularity (often <5%) and an abundance of extracellular mucin; and (3) the absence of relevant preclinical model systems such as cell lines or xenografts. Notably, none of the molecular alterations identified so far have directly impacted patient care and all resulted from candidate gene approaches.

Systematic genome-wide sequencing of many cancer types has recently revealed an extensive repertoire of cancer-driving genes and pathways. Such studies have led to an increased understanding of tumor biology offering new opportunities for therapy and prognosis. Many cancers including CRC have been initially explored genome-wide by analyzing a small number of samples [14], with findings later borne out by larger-scale studies [15]. Here, we present the first comprehensive molecular profiling of MCP from appendiceal origin, an advanced stage of MNA with limited treatment options. We performed whole exome sequencing of 10 cases, nine low-grade and one high-grade, and validated the results in 19 additional cases including three primary LAMN. Our findings have immediate implications for clinical care and suggest opportunities to develop novel therapies targeting pathways driving both tumor growth and mucin production.

## Methods

### Specimens

Research on specimen from human MNA was approved by the Institutional Review Board of the University of California, San Diego. Before enrolling in the study, all patients gave informed consent. DNA was obtained from matched blood (normal DNA) or fresh frozen tumor tissue (tumor DNA) isolated by laser-capture micro-dissection (LCM) of tumor cells from MCP of appendiceal origin (nine low grade, one high grade samples from the discovery group - Additional file 1: Figure S1) or directly from the formalin fixed paraffin embedded (FFPE) slides (five high-grade MCP, 11 low-grade MCP, and three LAMN samples from the validation group).

### Sequencing

For the discovery group, we performed whole exome capture on 2.5 μg of genomic DNA from 10 normal samples or 500 ng of DNA from 10 matching LCM tumor samples using the Agilent SureSelect Human All Exon v4 capture kit. Each resulting library was sequenced using one-quarter of a lane of Illumina HiSeq 2000 for 2 × 100 cycles. The resulting reads were aligned to the human genome with BWA, refined with GATK, the somatic variants and copy numbers were called using Varscan. The raw sequencing reads are available at the NCBI short read archive (SRA067608). For the validation group, we detected the presence of low frequency mutations using ultra deep targeted sequencing of PCR amplicons from *KRAS* codons 12/13/146, *GNAS* codon 201/227, and *SMAD2* codon 465 on the Illumina HiSeq 2000. We also confirmed the presence of *KRAS* and *GNAS* mutations by digital droplet PCR assays specific to *KRAS* G12A/V/D and *GNAS* R201C/H (RainDance Technologies’ RainDrop dPCR system).

### Immunohistochemistry

Adjacent slides from FFPE blocks were stained for H&E or incubated with primary antibodies against Phospho-p44/42 MAPK (Erk1/2) (Thr202/Tyr204) or Phospho-(Ser/Thr) PKA Substrate (Cell Signaling Inc.). All staining was compared to normal appendix. The signal was scored by staining intensity (0 to 3+) and fraction of stained tumor cells (0% to 100%). The enzyme was considered active at a signal greater than 2 in more than 25% of tumor cells.

Complete material and methods are available as Supplemental Information (Additional file 2).

## Results and discussion

### Global mutational landscape

We identified 2,173 somatic mutations located in the sequenced regions across all 10 tumors (Additional file 3: Table S1) with a median of 54 non-silent mutations per tumor, a number similar to non-hypermutated colorectal cancers (CRC) [15]. Not surprisingly, the high-grade sample had the highest mutation rate (4.9 per Mbp). The median allelic fraction observed at somatically mutated positions was 0.43 (Additional file 1: Figure S2). Therefore the laser-capture microdissection successfully enriched for tumor cells up to 86%, ensuring sensitive mutation identification. The profile of substitutions showed a majority (55%) of C > T transitions, a common characteristic of CRCs, which suggests that similar mechanisms are involved in DNA mutagenesis and repair (Additional file 3: Table S2).

Of the somatic mutations identified, 693 predicted an altered protein sequence in 642 unique genes (Additional file 3: Table S3 and S4) of which 36 are mutated in two or more tumors. The altered genes significantly affected 20 known pathways (corrected *P* <0.01 - Additional file 3: Table S5), most of which represent common signaling pathways mutated in cancer, overlapping with Ras-PI3K-Akt, Wnt, TGFβ, or cAMP-PKA pathways. To determine the significance of each recurrently mutated gene, we measured their false recurrence rate (FRR) (Additional file 2). Of the 36 recurrently mutated genes, 25 were considered significant (FRR <0.05, Table 1). The non-significant genes contained expected false positives such as *TTN* and *MUC16* or *CSMD1*[16], highlighting the specificity of the FRR estimation. The significant genes included genes frequently mutated in CRC such as *KRAS* (mutated in N = 10/10), *SMAD2* (2/10), and *FAT4* (3/10), as well as genes mutated in other cancers such as *GNAS* (9/10). Ten of the 25 remaining genes could be linked to oncogenesis, however with a lesser degree of certainty. Surprisingly, the most frequently mutated genes in CRC, *APC* and *TP53,* were not recurrently mutated in the 10 MNAs analyzed, suggesting distinct oncogenic processes.

**Table 1:** Genes recurrently mutated in MNA

**Gene**	**N. MNA mutated**	**FRR**^**a**^	**Fraction of samples mutated (MNA | CRC**^**b**^**)**	**Link to oncogenesis**
*KRAS*	10	**0**	1 | **0.42**	Cancer Gene Census: pancreatic, colorectal, lung
*GNAS*	9	**0**	0.9 | 0.06	Cancer Gene Census: pituitary tumors
*POM121L12*	2	**0**	0.2 | 0.02	
*DOK6*	2	**0**	0.2 | 0.02	RET signaling
*IRX6*	2	**0.001**	0.2 | 0.02	Homeobox TF neurogenesis
*KRT37*	2	**0.001**	0.2 | 0.02	
*EEF1A1*	2	**0.003**	0.2 | 0.01	Proto-oncogene of PTI1: Prost. Tumor Inducing 1
*CRY2*	2	**0.003**	0.2 | 0.01	
*SMAD2*	2	**0.004**	0.2 | **0.04**	TFGβ pathway
*SNTG1*	2	**0.004**	0.2 | 0.02	
*OCA2*	2	**0.007**	0.2 | 0.03	
*EPHA10*	2	**0.007**	0.2 | 0.02	Receptor for EPH pathway
*ZNF512B*	2	**0.008**	0.2 | 0.03	
*PTCHD3*	2	**0.009**	0.2 | 0.04	Homologous to PTCH1: Shh receptor
*ANKRD24*	2	**0.009**	0.2 | 0.02	
*DCLK1*	2	**0.011**	0.2 | 0.05	Intestinal tumor stem cell marker
*PCDH10*	2	**0.011**	0.2 | 0.09	Silenced in multiple epithelial cancers
*MTIF2*	2	**0.014**	0.2 | 0.02	
*ITGA11*	2	**0.014**	0.2 | 0.02	
*COL5A3*	2	**0.014**	0.2 | 0.04	
*PCDH17*	2	**0.015**	0.2 | 0.08	Mutated and silenced in GI cancer
*CNTNAP2*	2	**0.018**	0.2 | 0.06	Tumor suppressor in Gliomas
*TRPS1*	2	**0.022**	0.2 | 0.09	GATA factor involved in EMT in breast cancer
*ABCA7*	2	**0.04**	0.2 | 0.06	
*FAT4*	3	**0.045**	0.3 | **0.2**	Putative receptor of Hippo pathway
*DOCK3*	2	0.061	NA	
*ZNF469*	2	0.067	NA	
*SPTA1*	2	0.068	NA	
*COL6A3*	2	0.089	NA	
*LAMA1*	2	0.105	NA	
*APOB*	2	0.261	NA	
*CSMD1*	2	0.305	NA	
*MUC16*	2	0.659	NA	
*TTN*	2	0.992	NA	

^a^False recurrence rate (FRR) <0.05 are in bold.

^b^MutSig significant (*P* <0.01).

**Figure 1:**
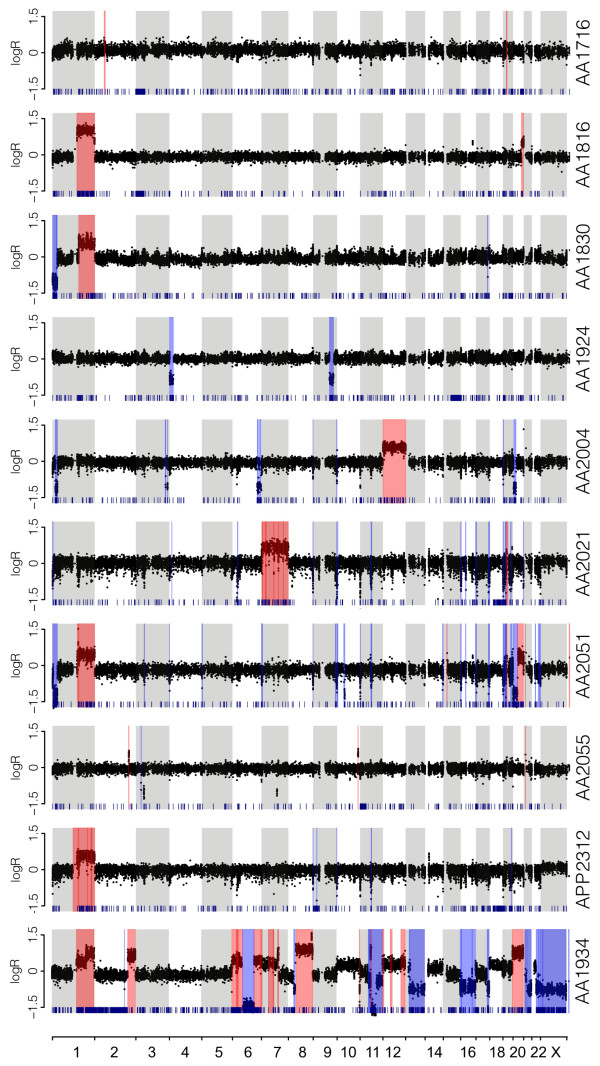
**Genome-wide copy number analysis.** The log2 ratio of coverage between tumor and normal is indicated for all targeted exons (black dots) along each chromosome arranged in linear order (alternate grey/white shade). The inferred segments larger than 1 Mb are indicated as amplified (red) or deleted (blue). Loss of heterozygosity at germline SNVs is indicated by a dark blue tick at the bottom of each panel. The top nine panels correspond to low-grade MCPs and the bottom panel to high-grade MCP.

Using the differences in coverage depth between tumor and normal DNA, as well as evidence of loss of heterozygosity, we were able to identify large (>1 Mb) copy number aberrations (CNAs) (Figure 1). CNAs in the low-grade MCPs represented a smaller portion of the genome than for the high-grade MCP (median 138 Mb *vs*. 1.1 Gb - Additional file 3: Table S6), suggesting a greater chromosomal instability in high-grade tumors. An analysis at the level of the chromosome arm and cytobands revealed several recurrent CNAs (Additional file 3: Table S7 to S9) among which the gain of 1q (amplified in N = 5 tumors), 20q (N = 2), 12p13 (N = 2), and 19p12 (N = 3) as well as the loss of 1p36 (N = 3), 20p12 (N = 2), or 16q24 (N = 3). Gain of 1q, 20q or 12p13 as well as loss of 1q36 or 20p12 are also frequent in CRC [15]. In contrast, CNAs present in more than 50% of CRC tumors, such as amplification of chromosomes 7, 8, or 13, loss of 8p or 18, were absent or only seen once in MNA. Notably, none of the amplified large chromosomal segments showed a ploidy higher than 4, and we identified only two focal deletions and one focal amplification, spanning *ST14*, *p15/p16*, and *CCND1* genes, respectively, in three independent samples (Additional file 3: Table S10). This suggests that high and recurrent copy number aberrations are uncommon and unlikely to drive MNA progression. Therefore both the sequencing and the copy number analysis demonstrate that while MNAs may share some somatic mutations with CRC, there are clear genetic differences between these cancer types.

### *GNAS* and *KRAS* mutations in low-grade MCP

**Figure 2:**
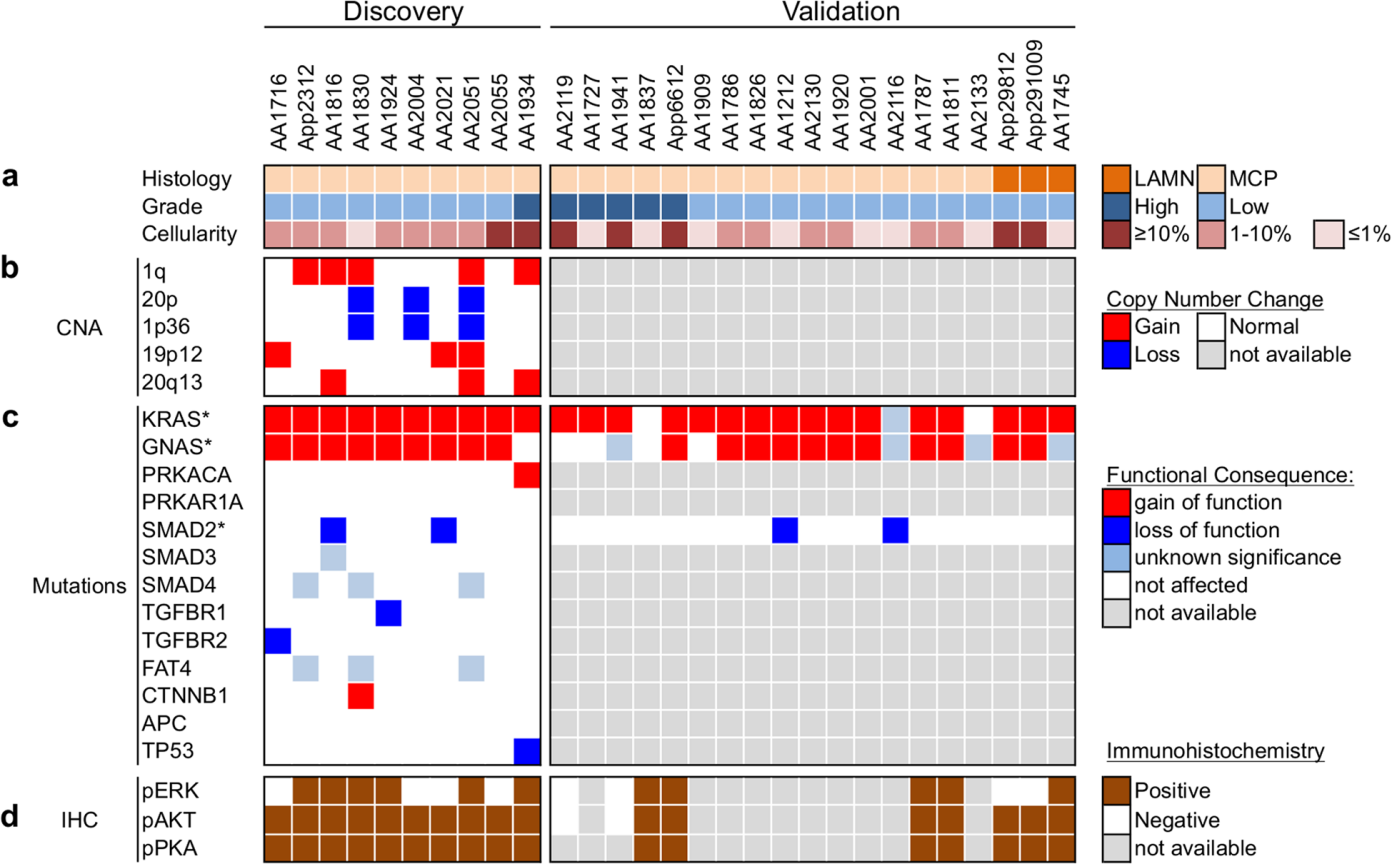
**The MNA mutational landscape.** Samples from 19 MNA patients were assessed for histopathology and cellularity **(a)**, analyzed for somatic mutations (Additional file 3: Tables S4 and S11) **(b)** or copy number aberrations (Additionalfile 3: Tables S7-S9) **(c)** as identified by exome sequencing using the discovery group (10 MCP samples - left side) or as validated by digital PCR and deep sequencing (19 samples - right side). **(d)** The levels of pErk, pAkt, and phosphorylated PKA substrates were measured by immunohistochemistry and determined to be positive based on signal strength and fraction of positive cells (Methods). (*) Only expected codons were investigated in the validation group. Low cellularity may impact the sensitivity of the validation.

**Figure 3:**
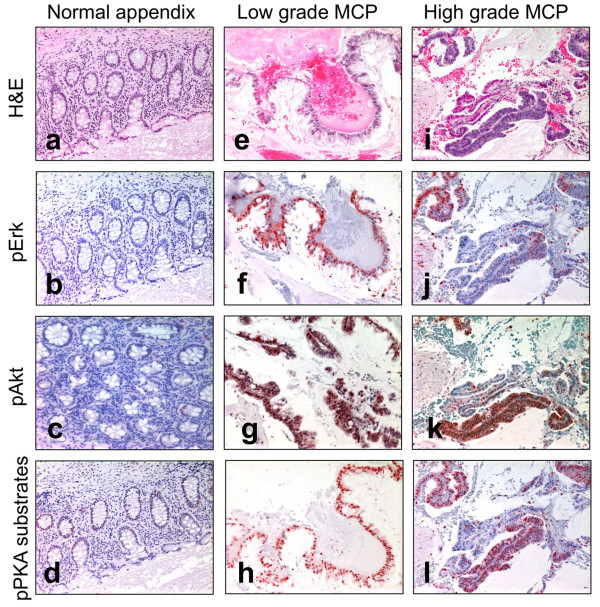
**Immunohistochemistry.** Normal appendix epithelium **(a-d)**, low-grade MCP **(e-h)**, high-grade MCP **(i-l)** samples were stained using H&E **(a, e, i)**, anti-pErk staining **(b, f, j)**, anti-pAkt staining **(c, g, k)**, and Anti-phospho-PKA substrates **(d, h, l)** on matched adjacent sections.

The most striking genetic alterations identified were the presence of both *GNAS* and *KRAS* mutations in every low-grade MCP. *KRAS* was mutated in all nine low-grade MCP samples, with three different missense alterations: G12V (N = 7), G12D (N = 1), and A146T (N = 1). The analysis of 14 additional samples, including three primary LAMN, by ultra-deep targeted sequencing revealed that *KRAS* was mutated in 91% (21/23) of low-grade MNAs (Figure 2 and Additional file 3: Table S11a). This incidence is significantly higher than observed in CRC [15] or in previous reports on MNA [5,7-10,13]. The less frequent *KRAS*-A146T mutation has been observed in approximately 5% of CRC [15] and increases RAS activity, mediating resistance to *EGFR* inhibitor gefitinib [17]. Immunostaining of 14 specimens (both low-grade MCP and LAMN), revealed frequent phosphorylation of Erk and Akt (Figures 2 and 3) as expected from constitutive RAS signaling predicted by oncogenic *KRAS* mutations. Finally, we observe, in one sample, the co-occurence of two *KRAS* mutations (G12V and G12D - Additional file 3: Table S17), indicating that different populations of KRAS-mutated cells can co-exist within one specimen, as previously observed in CRC [18].

*GNAS* encodes for the stimulatory G protein alpha subunit (Gαs). It was originally found to be mutated in pituitary tumors [19] and more recently in intraductal papillary mucinous neoplasms of the pancreas (IPMN), gastric, and intestinal adenomas, as well as CRC and low-grade mucinous neoplasm of the appendix (LAMN) [13,20-23]. We observed three types of *GNAS* mutations at two sites, R201C (N = 7), R201H (N = 1), and Q227H (N = 1), all of which are known to activate Gαs [24], resulting in increased cAMP levels and PKA activity. The analysis of 14 additional cases indicates that *GNAS* was mutated in 82% (19/23) of low-grade MCP or LAMN and immunostaining of phosphorylated PKA substrates in 14 low-grade samples reveals strong PKA activation in all cases (Figures 2 and 3), Finally, both *KRAS* and *GNAS* mutations detected in one low-grade MCP specimen were also identified in the matched primary LAMN and associated with the activation of Akt and PKA (Additional file 1: Figure S3) indicating, for this patient, the lack of major phenotypic evolution during the metastasis.

### PKA activity in high-grade MCP

The high-grade MCP analyzed by whole exome sequencing differs notably from low-grade MCPs by the larger number of chromosomal rearrangements, the presence of a *TP53* mutation and the absence of *GNAS* mutation. Using ultra deep targeted sequencing in five additional high-grade MCPs, we were able to detect a *GNAS* mutation only in one sample, suggesting that the *GNAS* mutational status is significantly different between low-grade and high-grade (21/23 *vs.* 1/6; *P* = 0.005; Additional file 3: Table S11 and Figure 2). This observation is consistent with the recent observation of *GNAS* mutations in LAMN but not in primary mucinous adenocarcinoma of the appendix [13]. By immuno-histology, we were able to observe the strong activation of PKA in 3/3 high-grade MCPs, even in absence of *GNAS* mutations (Figures 2 and 3). A close inspection of the mutations identified in the high-grade MCP sample from the discovery group revealed a W197S mutation in the catalytic subunit of PKA (encoded by *PRKACA*). A different substitution at the same residue is known to result in PKA constitutive activation [25].

### Additional mutated cancer genes

Other cancer genes were recurrently mutated in MNAs. Members of the TGFβ pathway *SMAD2*, *SMAD3*, *SMAD4*, *TGFBR1*, *and TGFBR2* were mutated in seven samples (Figure 2). All mutations are predicted to be loss of function (Additional file 3: Table S4). Interestingly both *SMAD2* mutations identified occur at Ser465 residue, thereby abolishing a phosphorylation site essential for Smad4 binding and propagation of TGFβ signaling [26]. Furthermore, both *SMAD2* mutations are homozygous due to a loss of heterozygosity of chromosome 18 (Additional file 1: Figure S4), suggesting a complete loss of function. The ultra deep targeted sequencing of 19 additional samples reveals deleterious mutations at Ser464 and Ser467 in two samples (Additional file 3: Table S11a), suggesting that overall, Smad2 activity is impaired in approximately 13% of MNAs. Similarly, both *TGFBR1/2* mutations are homozygous and *TGFBR1*-S241L has been identified in inherited diseases [27,28], suggesting a loss of function. Therefore, our analysis indicates that 7/10 MNAs may have impaired TGFβ signaling, a higher incidence than that observed in non-hypermutated CRC (24%). Genes in the Hippo pathway were also altered, affecting 4/10 MNAs through mutations in *FAT4*, *FAT3*, and *DSCH1*. Hippo pathway inactivation has been observed in cancer [29] including CRC in which both *FAT4* and *FAT2*, putative Hippo pathway receptors [30], are significantly mutated in 24% of the cases [15]. *FAT4* has been recently established as a tumor suppressor [31], generally mutated in less than 10% of epithelial cancers (Additional file 3: Table S12). Our results therefore suggest that the inactivation of genes in the Hippo pathway may contribute to MNA development in ways similar to CRC. Genes belonging to the Wnt pathway were mutated in 3/10 MNAs. Mutations in *WNT7A* and *WNT10A* are predicted to be deleterious (Additional file 3: Table S4) while *CTNNB1*-D32N is known to affect the ubiquitination recognition motif of beta-catenin resulting in increased protein stability [32]. This suggests that Wnt activation may promote oncogenesis in a subset of MNAs, despite the notable absence of *APC* alterations frequently observed in CRC. Finally, we observed non-synonymous mutations in other cancer-related genes such as *ATR*, *TP53* (DNA repair), *PIK3CA* (Akt signaling), *c-FOS* (Immediate early oncogene), *GRIN2B/2C* (Glutamate receptors) or *PKCB/D* (PKC subunits). These results, together with the recurrent mutated gene analysis (Table 1) suggest that several pathways may contribute to MNA oncogenesis. Importantly both mutations in DNA repair pathway genes *ATR* and *TP53* - with loss of heterozygosity - were identified in the high-grade MCP sample consistent with its higher mutation rate.

### MNA molecular relationship to colorectal cancer

Since it was first described in 1842 by Rokitansky, the site of origin and the underlying pathophysiology of tumors that most commonly give rise to MCP and PMP have been much debated. The disease classification has proven difficult owing to the varying histologic appearance and clinical behavior of MNA that metastasize to the peritoneal surfaces [2]. We present for the first time, a genome-wide mutational analysis of MCPs of appendiceal origin, which provides the genetic basis of this unusual disease. Our analysis reveals some genetic similarities between MNA and CRC, but many striking differences. Altered in less than half of CRC, *KRAS* mutations are present in 90% of MNAs. Other CRC-related genetic alterations include frequent amplification at chromosome 1q and loss at the 1q36 locus. This result suggests that *MCL1*, located on 1q21 and recently reported as amplified in MCP of appendiceal origin [33], is part of larger chromosomal gain. Its oncogenic role in MCP or MNA remains to be established. Mutations in the Wnt pathway, common in CRC, are also seen in MNA, but with the remarkable absence of *APC* mutations. Similarly, TGFβ is impaired in two cases by the *SMAD2* mutations at Serine 465, never observed in CRC [34]. Another striking difference between MNA and CRC is the absence of *TP53* mutations, except for a high-grade sample, consistent with the association of high-grade histology with increased chromosomal instability. In complement to our study, we also ordered the clinical sequencing of a 236-genes panel (Foundation One - Foundation Medicine, Cambridge, MA, USA) for seven additional patients diagnosed with high-grade MCP. The test includes genes such as *APC*, *TP53*, *KRAS*, *GNAS*, *CTNNB1*, *TGFBR2*, *SMAD2*, *SMAD4*, and *PKAR1A*. While no mutations were reported in *APC*, *SMAD2*, *CTNNB1*, or *PKAR1A*, the majority of patients carried mutations in *KRAS* (N = 4), *GNAS* (N = 1), *TP53* (N = 3), *TGFBR2* (N = 1), and *SMAD4* (N = 1), thus showing consistency with our initial findings. One explanation for the lower fraction of KRAS-mutated samples is the greater sensitivity of our research assay, involving laser capture microdissection, inclusion of a matched germline control and analytical optimization for low cellularity specimen. Owing to the rarity of MNA and the technical challenges with tissue collection and sample preparation, our study was conducted with a relatively limited number of samples and the investigation of additional samples will be important to determine the potential role TGFβ and Wnt signaling pathways in appendiceal tumorigenesis.

### Perspective on Ras and PKA pathway crosstalk

**Figure 4:**
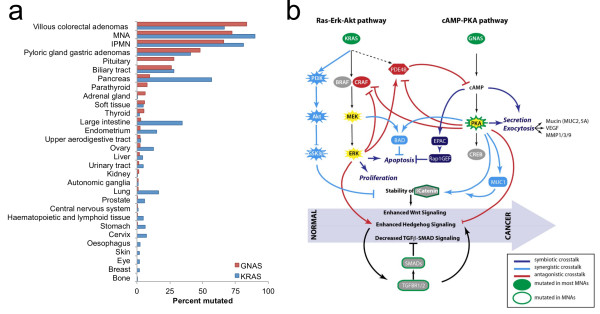
**Frequent and complex interplay between *KRAS *and *GNAS *in MNAs. (a)** High frequency of co-existing *KRAS* and *GNAS* mutations is uncommon in neoplastic lesions. Bar graphs represent the frequency of activating mutations in *KRAS* and *GNAS* oncogenes among various neoplasms reported in COSMIC (v63 database) or in IPMN [22] or MNA (this study). Only tissues with more than 10 evaluated samples are reported. **(b)** Simplified representation of the cross-talk between Ras and PKA pathway and the likely impact on MNA progression. Co-existing activating mutations in *KRAS* and *GNAS* can coordinately deregulate multiple oncogenic signaling pathways (Wnt, hedgehog, and TGFβ-SMAD) triggering initiation and progression of MNAs. Symbiotic (dark blue), synergistic (light blue), and antagonistic (dark red) signals triggered by mutations in *GNAS* and *KRAS* oncogenes, and possibly members of the TGFβ-SMAD pathway may all contribute to the metastatic progression of MNA.

In contrast to a previous report which lacked sensitivity and did not assess downstream pathway activity [13], our work formally establishes the universal activation of the PKA pathway in most MNAs, most often through activating mutations in *GNAS*. We observed that activating mutations in *GNAS* and *KRAS* co-exist in neoplasms at high frequency only in MNAs and pancreatic IPMN (Figure 4a). Interestingly, both IPMN and low-grade MNA are benign tumors that share important common clinico-pathologic characteristics such as slow-growth, invasiveness, and high production of mucin. In addition, when clinically localized to the organ of origin, they each have a favorable prognosis when treated with surgical resection. It is possible that the common clinico-pathological features are the direct consequence of the simultaneous activation of *KRAS* and *GNAS* within two major signaling pathways (Ras-PI3K-Akt and cAMP-PKA). The activation of these two pathways can be symbiotic and sustain cell growth (Figure 4b). Indeed, although *KRAS*-mutated cells are susceptible to apoptosis in low-glucose condition, this effect can be countered by high cAMP levels, increasing proliferation [35]. In addition, *GNAS-*mutated cells may not have proliferative advantage over normal cells [36] but are predicted to be more invasive by triggering regulated exocytosis of multiple MMPs, VEGF and mucins [37-40] and transcriptional upregulation of *MUC1* oncogene [41]. Thus, this suggests that each mutation depends on the other to gain some advantageous phenotype, balancing proliferation, and invasive capacity. Additional interactions in the two pathways may explain the relatively indolent course observed in both MNA and IPMN. Indeed the Ras-PI3K-Akt pathway activates *PDE4B*, a potent scavenger of cAMP [42], thus mitigating the effect of *GNAS* mutations located upstream in the pathway. Interestingly, we found that a high-grade MCP lacking *GNAS* mutations carries instead an activating mutation in PKA. Such tumors would be insensitive to Ras-PI3K-Akt mediated activation of PDE4B and degradation of cAMP. This effect would allow the synergy between RAS and cAMP pathways to impart a higher-grade histology. Furthermore, cAMP is known to inhibit Ras-Raf-Erk signaling [43]. This antagonism can be observed in our IHC experiment (Figure 2) where 11/19 and 19/19 MNAs display activated Erk and Akt, respectively, suggesting that the Ras- PI3K-Akt pathway is more consistently activated than the Ras-Raf-Erk pathway. We identified additional potential crosstalk between the two pathways, both synergetic and antagonistic (Figure 4b), suggesting areas of future investigation to determine their importance in MNA etiology. The absence of recurrent *GNAS* mutations in high-grade tumors also suggests that, in most cases, they do not progress from a low-grade lesion. The only high grade MCP case also mutated for *GNAS* showed additional low-grade lesions in different areas of the peritoneal cavity. The specimen studied may have contained a portion of low grade MCP not seen in the diagnostic section, or alternatively, both types of lesions may have a putative common origin in this patient. The presence of *KRAS* alterations in both high-grade and low-grade tumors suggests that the *KRAS* mutation occurs earlier in the course of tumorigenesis. Finally, lesions acquiring *GNAS* mutations seem to ultimately result in low-grade tumors, while activation of the PKA signaling via alternative mechanisms, accompanied by additional mutations such as *TP53*, seems to be characteristic of high-grade tumors.

### Clinical implications

*GNAS* mutations have also been recently reported in precancerous/benign lesions of the digestive tract: villous adenomas in the stomach and in the colon [20,23]. Interestingly, villous architecture and high mucin secretion are a common characteristic of these tumor types. The malignant potential of villous adenomas of the appendix is well-documented and it is estimated that approximately 40% to 66% of non-invasive adenomas may eventually transform into carcinoma in situ [44]. Thus, it is tempting to speculate that MNAs may originate from villous adenomas of the appendix. In contrast to colorectal villous adenomas, mucin generated by MNA would accumulate locally in the small lumen of the appendix, leading to rupture into the peritoneal cavity and increased risk of MCP.

Our findings also have some immediate implications for the management of patients with MCP secondary to MNA that is not amenable to surgical cytoreduction. *KRAS* testing done via CLIA approved methods has yielded mutation rates in the range of 50% to 60%, considerably lower than what we observed in this study. It is not surprising that current diagnostic assays underestimate the incidence of *KRAS* mutations given the hypocellular nature of metastatic lesions from MCP. False negative results have profound clinical implications as many MNA patients are subject to therapy with EGFR monoclonal antibodies which are ineffective and potentially harmful in the presence of mutant *KRAS*. Our findings also raise the potential for novel targeted approaches to MNA. The universal presence of oncogenic *KRAS* mutations leading to phosphorylation of Erk, as well as the fact that MEK-ERK signaling can activate transcription of *MUC2*, suggests that MEK inhibitors would be logical agents to explore in this disease. Similarly, given the consistent activation of Akt in MNA, targeting PI3K/AKT signaling may also have clinical benefits [45]. Agents targeting the cAMP-PKA pathway would also be sensible choices given the deregulation of this pathway in nearly all samples. Given that the production and secretion of mucin accounts for much of the morbidity from this tumor type, even therapies that act solely to reduce mucin production and secretion alone could have profound clinical value. Finally, agents targeting TGFβ and Wnt signaling may provide therapeutic opportunities in selected cases. The current lack of suitable preclinical models of MNA make proof of concept studies challenging, but the information gained here may guide development of such models and encourage further research on MNAs.

## Conclusions

The genome-wide mutational analysis of mucinous carcinomatosis peritonei of appendiceal origin reveals genetic alterations distinct from colorectal cancer. Mutations in both Ras and PKA pathways, which are tightly interacting, are consistent with the unique pathophysiology of the disease: a slow progressing neoplasm accompanied by important secretion of mucin. Additionally, the lower prevalence of *GNAS* mutations in high-grade lesions indicates that they do not progress from low-grade. Finally, the very frequent activation of Ras and PKA pathways, as well as sporadic alterations in TGFβ and Wnt signaling pathways suggest, for the first time, that targeted therapies may have important benefits for these patients, which are currently primarily treated with surgery and chemotherapy.

## Abbreviations

CRC: Colorectal cancer; FFPE: Formalin fixed paraffin embedded; FRR: False recurrence rate; LAMN: Low-grade appendix mucinous neoplasm; MCP: Mucinous carcinomatosis peritonei; MNA: Mucinous neoplasm of the appendix; PMP: Pseudomyxoma peritonei.

## Competing interests

MLS is an employee from RainDance Technologies. The remaining authors declare that they have no competing interests.

## Authors’ contributions

HA, MLB, KJ, YD, AA, and MLS generated the data. HA, SY, and OH analyzed the data. HA, MLB, ESM, RBS, MRP, and AML collected and analyzed the tissue specimen. HA, KAF, AML, and OH designed the study. HA, PG, KAF, AML, and OH wrote the manuscript. All authors read and approved the final manuscript.

## Supplementary Material

Additional file 1 Supplemental Figures S1 to S6. Click here for file

Additional file 2 Supplemental Methods. Click here for file

Additional file 3 Supplemental Tables S1 to S17. Click here for file
