# Supplementary material for: Genome-wide mutational landscape of mucinous carcinomatosis peritonei of appendiceal origin
Source: Genome Med. 2014 May 29;6(5):43. doi: 10.1186/gm559 (PMC4062050; doi:10.1186/gm559)
Supplement: Additional file 1 — Supplemental Figures S1 to S6. [file gm559-S1.pdf]

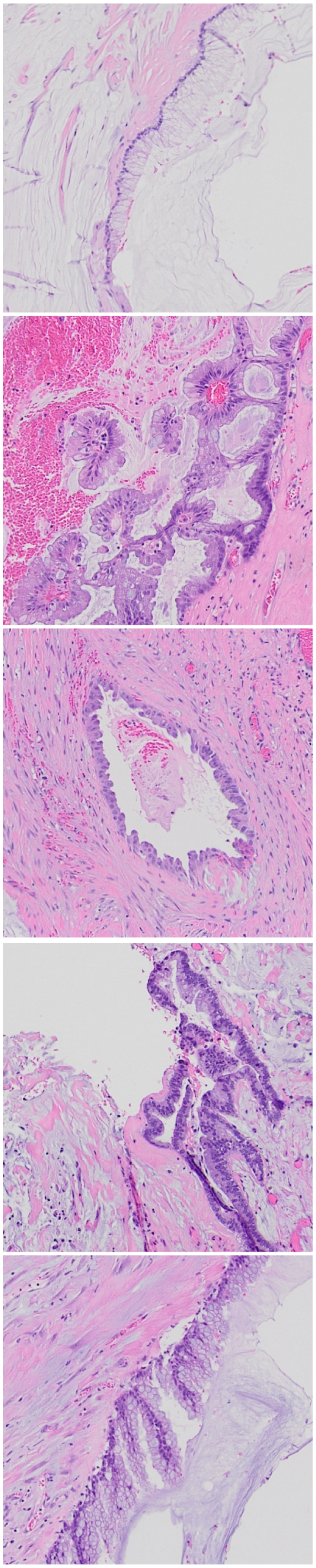

Patient-ID AA1716
Age 59
Gender Male
Site of origin Peritoneal metastasis
Grade Low-grade
Description A strip of neoplastic mucinous epithelium is seen with low grade cytologic and architectural features in a background of abundant extracellular mucin.

Patient-ID App2312
Age 64
Gender Female
Site of origin Peritoneal metastasis
Grade Low-grade
Description Papillary and glandular formations of neoplastic mucinous epithelium are seen with low grade cytologic and architectural features in a background of extracellular mucin and blood.

Patient-ID AA1816
Age 42
Gender Male
Site of origin Peritoneal metastasis
Grade Low-grade
Description A single glandular structure composed of neoplastic mucinous epithelium with low grade cytologic and architectureal features is seen embedded in fibrous tissue.

Patient-ID AA1830
Age 72
Gender Female
Site of origin Peritoneal metastasis
Grade Low-grade
Description Strips of neoplastic mucinous epithelium are seen with low grade cytologic and architectural features, associated with abundant extracellular mucin.

Patient-ID AA1924
Age 62
Gender Male
Site of origin Peritoneal metastasis
Grade Low-grade
Description Strips and papillary formations of neoplastic mucinous epithelium are seen with low grade cytologic and architectural features, associated with abundant extracellular mucin.

Figure S1 […]

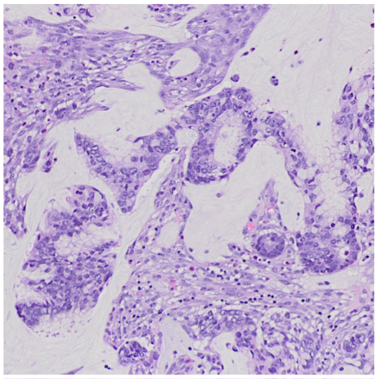

Patient-ID AA1934
Age 50
Gender Female
Site of origin Peritoneal metastasis
Grade High-grade
Description Crowded aggregates of floating neoplastic mucinous epithelium are seen with complex architecture and high grade cytologic features.

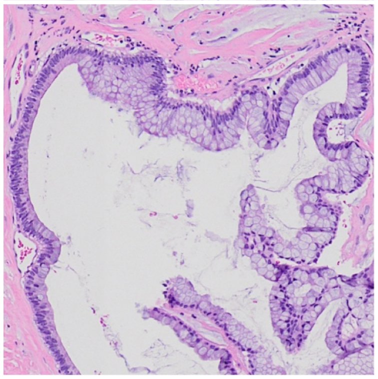

Patient-ID AA2004
Age 34
Gender Female
Site of origin Peritoneal metastasis
Grade Low-grade
Description Strips of neoplastic mucinous epithelium are seen with low grade cytologic and architectural features, associated with abundant extracellular mucin.

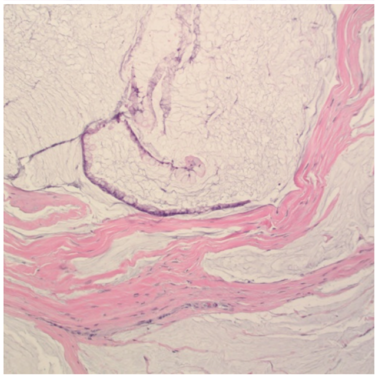

Patient-ID AA2021
Age 45
Gender Female
Site of origin Peritoneal metastasis
Grade Low-grade
Description Small strip of neoplastic mucinous epithelium with low grade cytologic and architectural features is seen associated with abundant extracellular mucin.

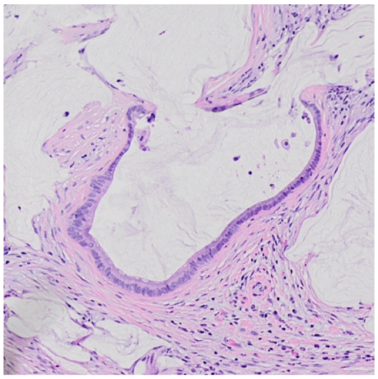

Patient-ID AA2051
Age 51
Gender Female
Site of origin Peritoneal metastasis
Grade Low-grade
Description A strip of neoplastic mucinous epithelium is seen with low grade cytologic and architectural features, associated with abundant extracellular mucin.

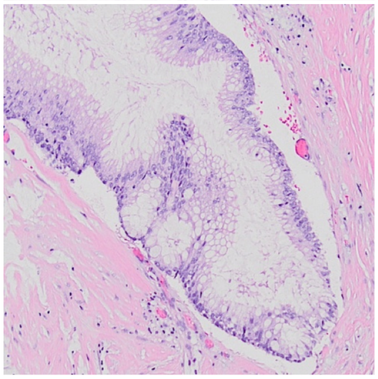

Patient-ID AA2055
Age 54
Gender Male
Site of origin Peritoneal metastasis
Grade Low-grade
Description A strip of neoplastic mucinous epithelium is seen with low grade cytologic and architectural features, associated with abundant extracellular mucin.

Figure S1 […]

Figure S1: H&E stained histology sections from the discovery group. The sections presented were selected as representing the diagnosis of the patient, including histological grade, and containing a high fraction of tumor cells for visualization purpose. For each section, the location, grade and description of the area displayed are indicated.

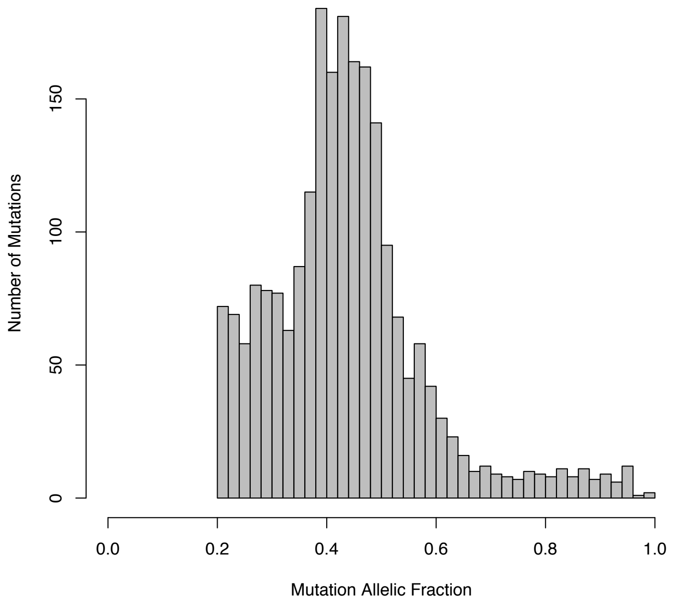

Figure S2: Distribution of the Mutant Allelic Fraction at all positions determined to be somatically mutated by whole exome sequencing of 10 MCPs.

a

								Blood			Tumor		
**Patient ID**	**Specimen**	**Gene**	**Mutation**	**Chr**	**Coord**	**Ref**	**Alt**	**Ref coverage**	**Alt Coverage**	**Allelic Fraction**	**Ref Coverage**	**Alt Coverage**	**Allelic Fraction**
AA1811	MCP	KRAS	G12V	chr12	25398284	C	A	198236	153	0.08%	95681	1227	1.27%
AA1811	LMNA	KRAS	G12V	chr12	25398284	C	A	198239	153	0.08%	180783	4488	2.42%
AA1811	MCP	GNAS	R201C	chr20	57484420	C	T	177522	218	0.12%	117171	4074	3.36%
AA1811	LMNA	GNAS	R201C	chr20	57484420	C	T	177524	218	0.12%	249506	8961	3.47%

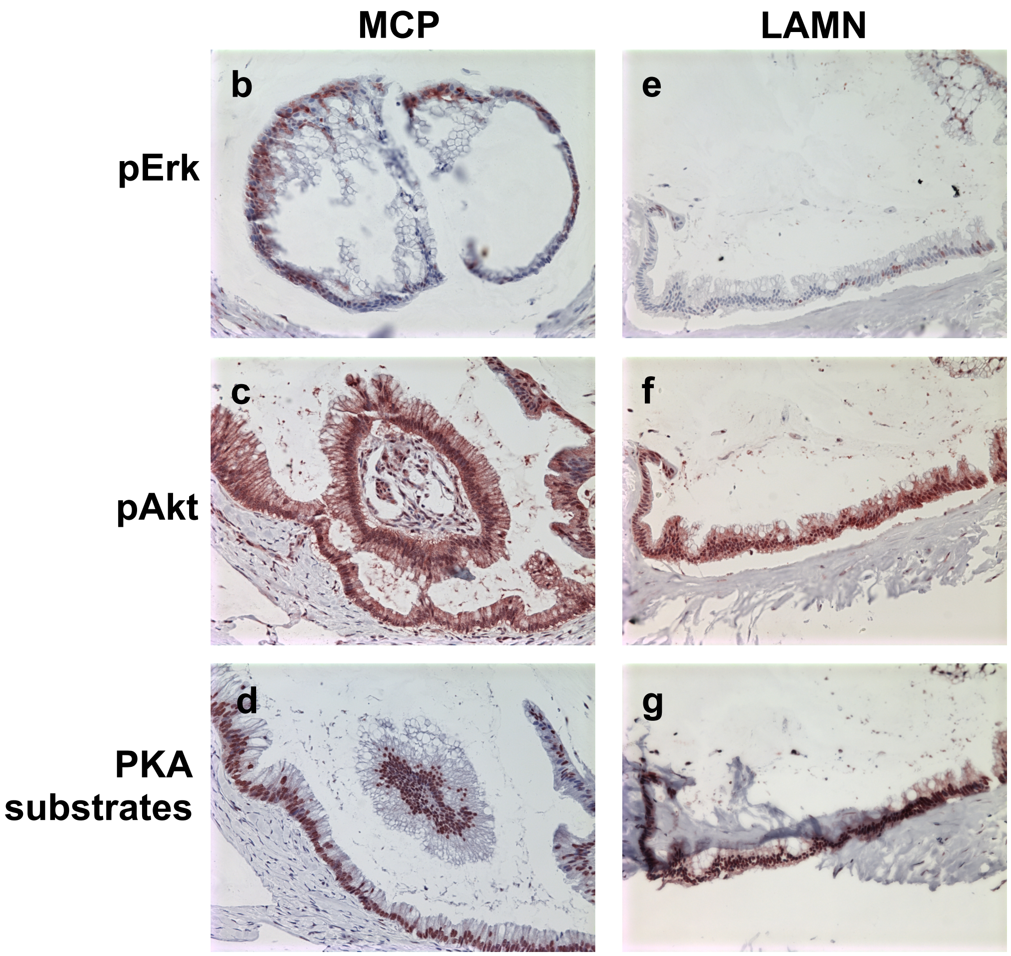

Figure S3: Analysis of matched low-grade MCP and primary LAMN from patient AA1811. (a) Summary table presenting the coverage and mutant allelic fraction observed at KRAS G12V and GNAS R201C mutations in both specimen. **(b-g) Immunohistochemistry.** The low grade MCP **(b-d)** and matched primary LAMN **(e-f)** sections were stained using Anti-pErk staining (**b**,**e**), Anti-pAkt staining (**c**,**f**) and Anti-phospho-PKA substrates (**d**,**g**).

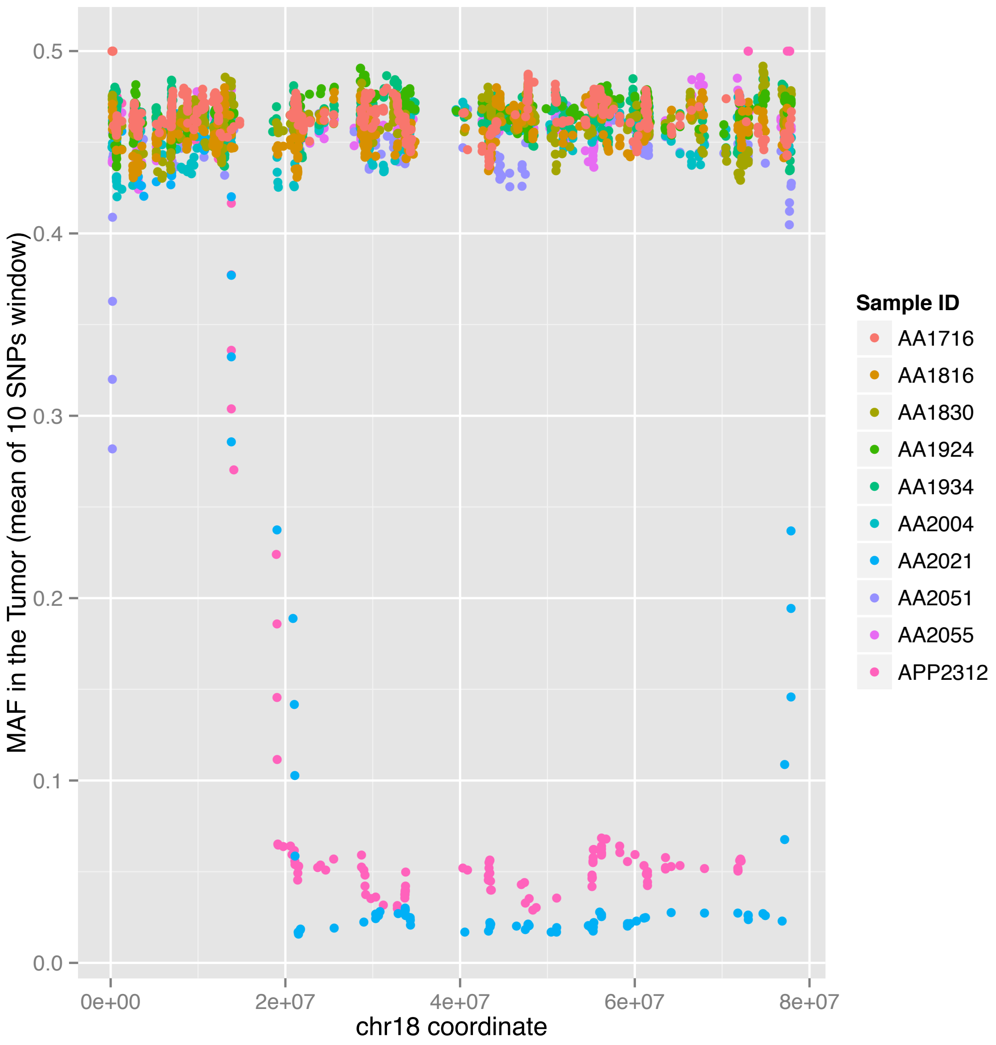

Figure S4: Evidence of chromosome 18 Loss of Heterozygosity. The minor allele frequency observed in the tumor at chromosome 18 heterozygous SNPs. We selected SNPs with Normal Allele frequency between 0.3 and 0.7 and cumulative coverage in normal and tumor greater than 100 fold.

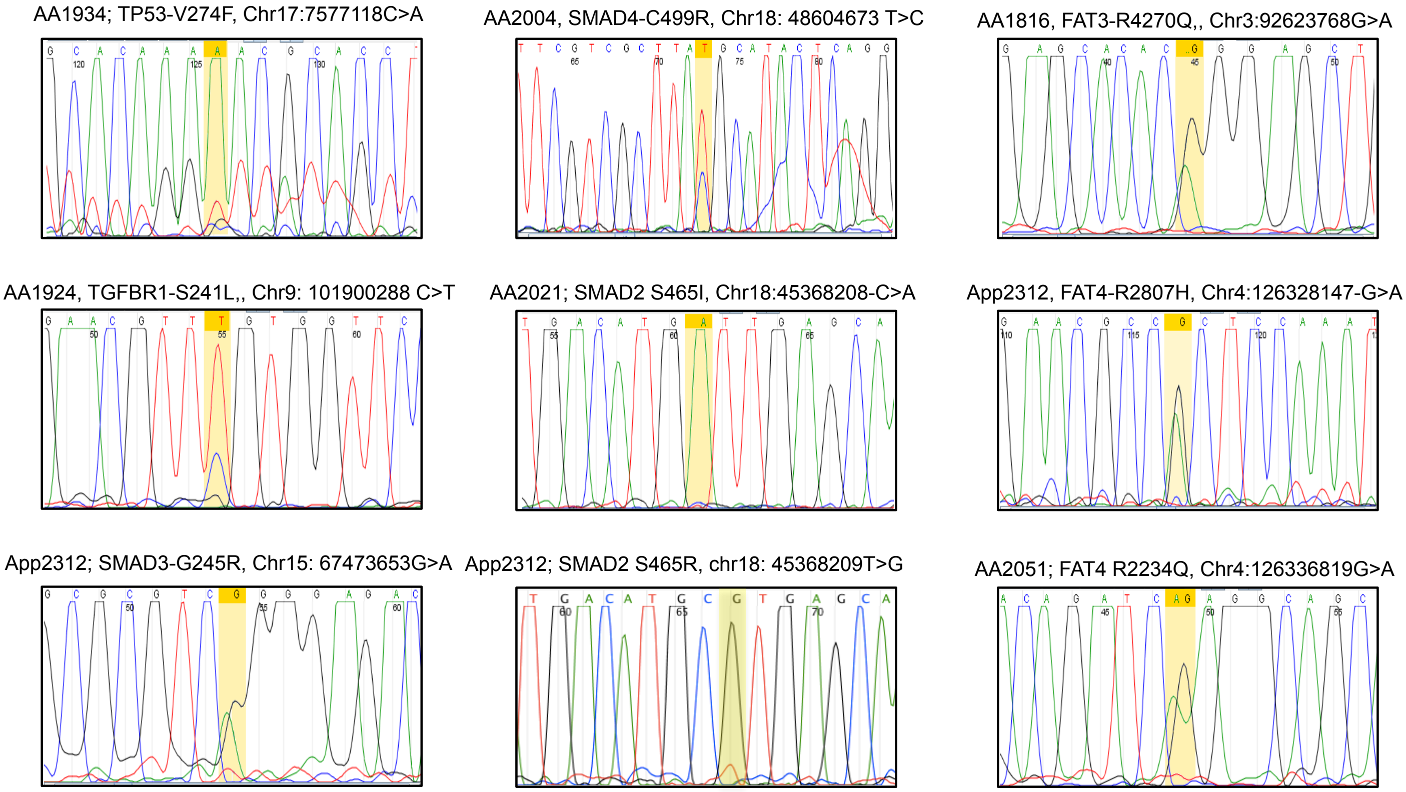

Figure S5: Confirmation by Sanger sequencing of the coding mutations identified in the discovery group in the genes TP53, FAT3/4, SMAD2/3/4 and TFBR1/2. Above each panel the samples, the coding mutations as well as nucleic acid substitution are indicated.

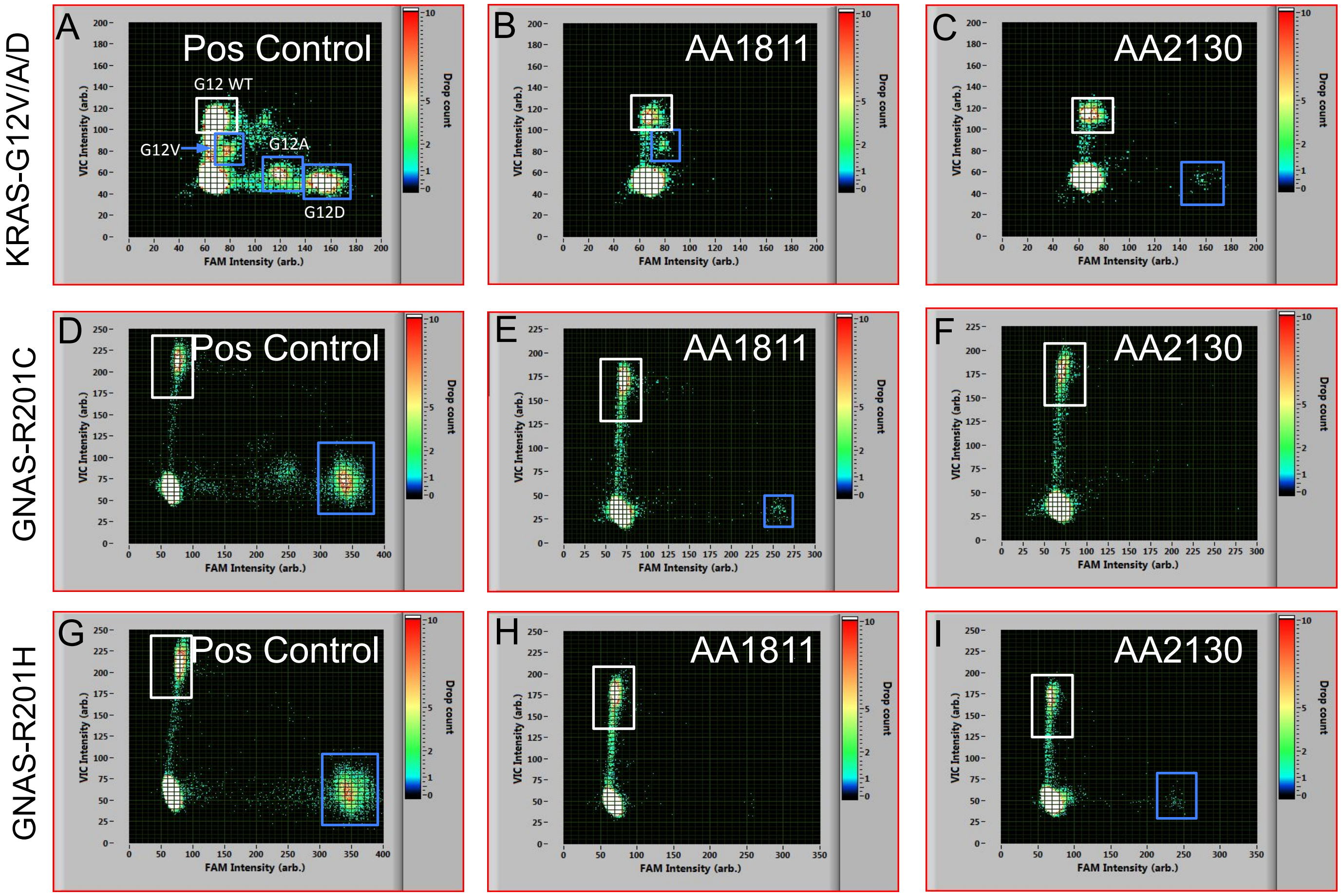

Figure S6: Digital Droplet PCR. Representative 2D histograms displaying the distribution of droplets (colored dots) as a function of the intensity in WT probe fluorescence (y axis) versus mutant probe fluorescence (x axis). Clusters of droplets are framed in white (WT) or blue (mutant) **(A-C)** KRAS-G12V/A/D assay, representing a positive control containing three mutations (A) along a G12V positive (B) and a G12D positive sample (C). **(D-F)** GNAS-R201C assay representing a positive control (D: AA2051 after LMD) along with positive (E) and negative (F) samples. **(G-I)** GNAS-R201H assay representing a positive control (G: AA2004 after LMD) along with negative (H) and positive (I) samples.
