# Supplementary material for: Genome-wide mutational landscape of mucinous carcinomatosis peritonei of appendiceal origin
Source: Genome Med. 2014 May 29;6(5):43. doi: 10.1186/gm559 (PMC4062050; doi:10.1186/gm559)
Supplement: Additional file 2 — Supplemental Methods. [file gm559-S2.docx]

**Supplemental Methods**

**Specimen Preparation**

*Sample collection*: The use of human Mucinous Neoplasms of the Appendix was approved by the Institutional Review Board of the University of California, San Diego. Before enrolling in the study, patients had to give informed consent. Blood samples used for germline DNA extraction was collected before surgery. The 29 tumor samples used in the study (10 in discovery, 19 in validation) were collected during surgical tumor resection. The fresh samples were kept on ice then fixed in 10% formalin, embedded in paraffin and H&E-stained for evaluation by a trained pathologist. For the 10 MCPs of appendiceal origin used in the discovery group (9 low-grade, 1 high-grade), a small part of the fresh sample was placed into 2x2x2 cm wells (Tissue Tek, Miles Scientific) and covered with OCT before flash freezing (Table S13). Notably, the samples selected for the discovery group had the highest tumor cell content to facilitate the laser capture microdissection. The 19 samples from the validation group have lower cellularity and comprised 5 high-grade MCP, 11 low-grade MCP and 3 LAMN. All DNA specimens from the validation group were extracted from formalin fixed blocks after histological examination.

*Histological Examination:* Hematoxylin and eosin-stained slides from routinely processed, formalin-fixed tissue sections from appendiceal and peritoneal specimens were reviewed. Mucinous tumors of the appendix were classified as either low-grade appendiceal mucinous neoplasm (LAMN) or mucinous adenocarcinoma, using the criteria given in the WHO Classification of Tumours of the Digestive System, 4^th^ edition (1). Briefly, LAMN is characterized by neoplastic mucinous epithelial cells with low-grade cytologic features growing in villous, serrated, or villous patterns, and associated with abundant extracellular mucin. Mucin extravasation into the wall of the appendix is commonly seen in LAMN, but true tissue invasion with associated desmoplasia is absent. Mucinous adenocarcinoma of the appendix is characterized by an invasive growth pattern with desmoplastic stroma and variable levels of cytologic dysplasia.

Peritoneal specimens were evaluated for the presence of extracellular mucin and neoplastic mucinous epithelium. Cases with mucinous carcinomatosis of the peritoneum were classified as either low-grade or high-grade, again using the criteria given in the WHO Classification of Tumours of the Digestive System, 4^th^ edition(1) (Discovery Group: Figure S1). Briefly, low-grade mucinous peritoneal carcinomatosis shows architecturally simple strips or small floating aggregates of neoplastic mucinous epithelium with bland cytologic features and overall low cellularity. In contrast, high-grade mucinous peritoneal carcinomatosis is characterized by more complex architectural patterns (especially cribriforming), tissue invasion with desmoplasia, high-grade cytologic features, and increased cellularity. The appendiceal origin of the primary lesion was verified at surgery by examination and resection of the appendix, or by inspection of the medical records for patients operated at a different hospital.

*Tumor cell enrichment by Laser-Capture Microdissection (LMD)***:** The OCT-frozen samples were kryo-sectioned at a thickness of 15 μm per section. The sections were attached on membrane coated microscopy slides (MMI®, Switzerland, Product Nr.:50103) and H&E-stained as follows: 95% Ethanol for 30 s, 70% Ethanol for 15 s, Water for 15 s, Hematoxylin for 5 s, Water rinse 2 x 15 s, Bluing solution for 5 s, 70% Ethanol for 15 s, Eosin for 5 s, 95% Ethanol for 15 s and 100% Ethanol for 2 x15 s. After staining, the samples were overnight-drained in an incubator at 56ºC. LMD was performed on these sections on a Leica LMD 7000-system at a magnification of 100x. The number of dissected cells was estimated from the dissected area, aiming for >100.000 cells per sample. A median number of 24 sections per sample (range: 16 to 38) was used for LMD.

*DNA isolation:* Tumor DNA from the discovery samples was extracted from LMD cells by using the QIAmp DNA Micro kit (Qiagen®) according to the manufacturer´s instructions after an overnight incubation of the samples in 180 µl Buffer ATL and 20 µl Proteinase K. The DNA concentration was determined by fluorometry (Qubit®, Life Technologies). The germline DNA was extracted from 100 µl buffy coats by using the DNeasy Blood and Tissue kit (Qiagen®) according to the manufacturer´s instructions. For each validation sample, the DNA was extracted from four 20 µm slides of formalin fixed tissue. DNA isolation was performed with the QIAmp DNA FFPE Tissue kit (Qiagen®) according to the manufacturer´s instructions.

**Exome Capture and Library Preparation**

The sequencing libraries were prepared and captured using SureSelect Human All Exon V4 kit (Agilent Technologies) following the manufacturer’s instructions. Briefly, 500 ng tumor DNA or 2.5 µg germline DNA from each sample was fragmented by Adaptive Focused Acoustics (E220 Focused Ultrasonicator, Covaris, Woburn, Massachusetts) to produce an average fragment size of ~175 base pairs. Fragmented DNA was purified using the Agencourt AMPure XP beads (Beckman Coulter, Fullerton, CA, USA). The quality of the fragmentation and purification was assessed with the Agilent 2100 Bioanalyzer. The fragment ends were repaired and adaptors were ligated to the fragments. The resulting DNA library was amplified by using manufacturer’s recommended PCR conditions: 2’ at 98°C followed by 6 cycles of (98°C 30”; 65°C 30”; 72°C 1’) finished by 10’ at 72°C. 500 ng of each library was captured by solution hybridization to biotinylated RNA library baits for 48 hrs at 65ºC. Bound genomic DNA was purified with streptavidin coated magnetic Dynabeads (Invitrogen, Carlsbad, CA) and further amplified to add barcoding adapters using manufacturer’s recommended PCR conditions: 2’ at 98°C followed by 12 cycles of (98°C 30”; 57°C 30”; 72°C 1’) finished by 10’ at 72°C.

**Exome Sequencing and Analysis**

Sequencing was performed using the Illumina HiSeq 2000 system, generating 100bp paired-end reads. All raw 100bp paired-end reads were aligned to the human genome reference sequence (hg19) using BWA v0.5.9-r16 (2) with default parameters for paired-end reads except for seed length set to 35. Aligned reads were realigned using GATK’s (3) IndelRealigner v 1.6-5-g557da77 combining all reads from the same patients and subsequently splitting them. Duplicate reads were removed using Picard Tools v 1.65 MarkDuplicates. Finally the GATK’s TableRecalibration tool was used to recalibrate the reads’ base quality scores. Table S14 presents the summary statistics of the sequencing. The sequencing data is publically available via the NCBI Short Read Archive (SRA067608).

*Variant Calling***:** We used VarScan2 v 2.3 (4) to compare the tumor to the normal sample and identify, for each patient, single nucleotide variants (SNVs) and small insertions and deletions (indels) that are: 1) inherited (germline variants); 2) acquired in the tumor (somatic mutations) as well as variants resulting from a loss of heterozygosity (LOH) or of unknown status. The required pileup files for VarScan2 were generated using SAMTools mpileup v 0.1.18 with default parameters except for –q 5, -Q 0, -d 50000, and -B. We used the default parameters for filtering variants except changing the tumor, normal, and combined minimum coverage to 10X each, minimum mutant allele frequency of 0.1, and minimum average quality score to 17. We then applied additional filtering steps. 1) *Low quality indels:* somatic indels with <10X coverage depth or fewer than 3 supporting reads or with more than 5% frequency in the germline are removed. 2) *VarScan default filters:* 2a) Variant within 3bp of an indel, 2b) clustering SNV: ≥3 SNVs located within 10bp, 2c) less than 10% allelic frequency. 3) *Low quality somatic variants:* Somatic variants with Varscan Fisher p-value<0.05 or with >5% alternate allele in the normal DNA (SNVs) or any alternate allele in the normal (indels) are filtered. 4) *VarScan2 high quality filter:* We finally applied VarScan2’s fpfilter script to both germline and somatic variants. This procedure filters variants based on their read position, strand bias, variant reads, variant frequency, distance to 3’, homopolymer, mapping quality difference, read length difference, and mismatch quality sum difference.

*Variant Annotation:* Variants were queried against dbSNP135 to determine novel or known variants. Next we used snpEff (5) v. 2.0.5 in combination with GATK VariantAnnotator, both with default parameters, to identify the different the functional and impact on coding genes. We enriched this annotation by cross-referencing the list of variants to the dbNSFP database(6), which provides conservation (PhyloP), functional prediction (SIFT, PolyPhen and Mutaster), as well as Uniprot codon change information. Finally, we annotated the variants for presence in COSMIC v61 based on coordinate and genotype. Notably, we used COSMIC codon numbering when discordant numbering were reported between databases.

*Copy Number Calling***:** We used VarScan2 copy number to call copy number aberrations (CNAs) in matched tumor-normal samples. We applied default parameters except, no minimum base or mapping quality, and segment size min=50 bp and max=1,000bp of contiguous covered bases. We used DNACopy R package to merge and smooth segments using their log2 ratio. We used a minimum log2 ratio of 0.25 and maximum log2 ratio of -0.25 to call amplifications and deletions, respectively. Arm level gain and losses were called when affecting more than 50% of the chromosome arm. Similarly, cytoband level gain and losses were called when affecting more than 50% of the cytobands. Finally, focal gain and losses were defined as genomic segments Amplified or deleted more than 4 fold in the tumor, containing at least 3 exons amplified or deleted more than 4 fold in the tumor.

*False Recurrence Rate:* We assessed the rate of false recurrence of all recurrently mutated genes using a permutation method. We performed N=1000 iterations of the following procedure to determine what fraction of iterations a gene *g* was determined to be recurrently identified in *n* samples or more (*n*≥2). At each iteration, we performed Q queries (Q corresponding to the number of samples in the cohort) of the database of non-synonymous mutations in dbNSFP(6). At each query *q*, we select *N(q)* non-synonymous substitutions where $N\left( q \right)=\sum_{x_{i}} N_{x_{i}}(q)$, with $N_{x_{i}}(q)$ corresponding to the number of non-synonymous substitution of type *x_i_* in sample *q* with x_i_ $\in$ {A>C, A>T, A>G, C>A, C>T, C>G}. Therefore, at each iteration the queries are pseudo-random, following the substitution profile and mutation rate in each sample of the cohort. The FRR of gene *g* at recurrence level *n* is then determined by the fraction of iterations where gene *g* carries non-synonymous mutations in *q* queries (*q≥n).*

*Mutation confirmation:* Using Sanger sequencing, we confirmed the presence of 9 selected mutations in genes of low recurrence in MNA: TP53, SMAD2, SMAD3, SMAD4, TGFBR1, FAT3, and FAT4 in 7 MCP samples from the discovery group. We prepared 50 µl PCR reactions using 5 µl 10 high-fidelity-PCR buffer, 1 µl mM dNTP mixture (New England BioLabs), 2 µl 50 mM MgSO4, 0,2 µl Platinum Taq (Invitrogen, Carlsbad, CA), 2 µl Primer Pairs (10 µM – Table S15), 5 ng DNA template. The PCR reaction was carried out using an ABI thermocycler using standard PCR conditions (94°C 60” followed by 35 cycles of (94°C 30”; 55°C 30”; 68°C 30”) finished by 10’ at 68°C. The PCR products were purified using a MinElute PCR Purification Kit (Qiagen) and used for Sanger-sequencing using each PCR primer as sequencing primers (Retrogen Inc, San Diego CA). We inspected the chromatograms for presence of the mutations (Figure S5). We were able to confirm 9/9 of the mutations investigated, verifying that mutations detected by exome sequencing and analysis are real.

*Pathway analysis:* We used the Ingenuity IPA canonical pathway database (Ingenuity Inc Redwood city CA) containing 590 human canonical pathways to determine which pathways were significantly enriched in the set of mutated genes (Table S5). The Fisher exact p-value calculated was corrected for multiple testing using Benjamini-Hochberg procedure.

**Immunohistochemistry**

Sections were generated from the FFPE blocks embedding MCP or LAMN specimen received from pathology. Normal human appendix 5 µm paraffin slides were purchased (#4286, AbCAM Cambridge, MA) for use as a negative control. Deparaffinized 5 µm sections were incubated in Antigen Retrieval Solution for 20 minutes at 95^o^C, then treated for 15 minutes with 0.3% hydrogen peroxide to block endogenous peroxidase activity. The slides were blocked with either 10% normal goat or donkey serum + 5% BSA in TBS-0.1%Tween20 for 1 hour at room temperature. Slides were incubated in primary antibody diluted in 5% normal goat serum in TBS-Tween20 or SignalStain Antibody Diluent (Cell Signaling Technology Inc., Danvers, MA) overnight at 4^o^C. The primary antibodies used included: Phospho-(Ser/Thr) PKA Substrate (#9621, Cell Signaling Inc), Phospho-p44/42 MAPK (Erk1/2) (Thr202/Tyr204)(D13.14.4E)XP (#4370, Cell Signaling Inc) and Phosph-Akt (Thr308) (#PA1-14030, Thermo Scientific). Slides were then washed and incubated with ImmPRESS Reagent Anti-Rabbit IgG (Vector Laboratories) for 30 minutes at room temperature, followed by wash and incubation with AEC Peroxidase Substrate (Vector Laboratories, Burlingame CA) for 15-25 minutes. After a final wash, slides were counterstained with hematoxylin, and mounted with DAKO Mount-Quick Aqueous Mounting Medium. Immunohistochemical staining was evaluated using a semi-quantitative method. The relative intensity of the staining was determined for each sample on a scale of 0 to 3 (0-None, 1-Weak, 2-Intermediate, 3-Strong). Samples were determined to be positive if more than 25% of the tumor cells had a staining of 2 or higher.

**Deep Targeted Sequencing**:

*PCR amplification and Sequencing:* For validation *KRAS*, *GNAS* and *SMAD2* mutations, 50 ng of DNA from paraffin embedded tumor tissue, or from buffy coats (germline DNA) was amplified using Platinum Taq (Invitrogen). Primers were designed around the mutated locus and tailed with an Illumina specific sequence (Table S16). The PCR reaction was carried out using an ABI thermocycler using standard PCR conditions (94°C 30” followed by 35 cycles of (94°C 30”; 60°C 30”; 68°C 30”) finished by 10’ at 68°C. The PCR products were purified using a MinElute PCR Purification Kit (Qiagen). 10 ng of PCR product was then PCR amplified for 2’ at 94°C followed by 10 cycles of (94°C 30”; 56°C 30”; 68°C 1’) finished by 10’ at 68°C using Illumina indexF and indexR primers (Table S16). The PCR products were purified using a MinElute PCR Purification Kit and checked for size and quality using DNA 1000 Agilent Bioanalyzer chip. The PCR amplicons were then sequenced on an Illumina HiSeq 2000 using 2 X 100 Paired End (PE) sequencing and a concentration between 45 and 100 fmol of DNA, in combination with other experiments using different indices. The sequencing led to an average of 180,536 fold coverage depth per amplicon, across all amplicons and samples.

*Analysis:* We analyzed the data using the Mutascope v1.0 pipeline (7), with the following parameters: 1) The error rate distribution was obtained from a broader sequencing (519 PCR amplicons) of a normal blood DNA from AA2004, 2) the local realignment and fisher exact p-value were estimated using AA1837 FFPE tissue DNA as a control, 3) the default filters were used, with the exception of strand bias, which was ignored. For each variant locus, we calculated the consensus quality score using *vcf-merge* (8). The resulting variants were annotated using variant tools (9) and the dbNSFP (6), dbSNP and COSMIC databases. Only coding non-silent mutations were reported (Table S11).

**Digital Droplet PCR**

*Reaction Preparation*: Control human DNA (Promega #G3041) was fragmented to a mean size of 3000 bp using adaptive focused acoustic shearing (S220 -Covaris) or mechanical shearing (Nebulizer – Life Technologies). The DNA extracted from FFPE tissue slides was not subjected to fragmentation. The primers and the FAM or VIC fluorescent Taqman MGB probes (Table S16) were designed using Applied Biosystems’ Custom TaqMan Assay Design Tool. The KRAS assay mix was prepared using 900 µM forward and reverse KRAS primers, 200 nM VIC-labeled WT KRAS probe, 50nM FAM-labeled G12A probe and 150nM FAM-labeled G12D probe. Each of the three GNAS assay mixes was prepared using a 40x Master Mix containing forward and reverse PCR primers, VIC-labeled WT probe and FAM-labeled mutant probe. The PCR reaction was prepared in 25 µl final volume, using 12.5 µl TaqMan Genotyping Master Mix (Life Technologies), 0.5 µl of 10mM dUTP, 0.25 µl AmpErase UNG (Life Technologies), 2.5 µl Droplet Stabilizer (RainDance Technologies), 2.5 µl Assay Mix (see above) and 6.75 µl DNA template in water (variable amount between 56 ng and 650 ng).

*Digital droplet assay:* Droplets containing PCR reaction components were generated using a hydrodynamic flow-focusing microfluidic chip (RainDrop Source chip – RainDance Technologies), deposited into PCR tubes as 5 pL aqueous droplets suspended in inert fluorinated oil (REB Carrier Oil; RainDance Technologies), and subjected to PCR amplification in a thermal cycler (Mastercycler proS, Eppendorf) using the following conditions: *KRAS* assay: 2 min at 50°C then 10 min 95°C, and 44 cycles of 95°C for 15 s and 64°C for 1 min. *GNAS* assay: 2 min at 50°C and 10 min 95°C, 44 cycles of: 95°C, 15 s and 60°C, 1 min. After PCR completion, the emulsion was injected into a second microfluidic chip (RainDrop Sense chip – RainDance Technologies) for fluorescence measurement. Following 488 nm excitation, droplet fluorescence was detected through filters (FAM & VIC emission) with photomultiplier tubes recording fluorescence intensity (“height”) and duration (“width”). Spectral crosstalk-corrected data from each sample or control was converted to a 2-dimensional (FAM & VIC) histogram (electronic and photonic noise was removed, droplet data was width filtered and normalized to background fluorescence). Custom software (RainDrop Analyst -RainDance Technologies) was used to define graphical areas or “gates” and count the number of droplets within each gate (Figure S6). The fraction of mutant allele in the sample was determined by the ratio of mutant droplets to total positive droplets (Table S17).

**Supplemental References**

1. Bosman FT, Carneiro F, for Research on Cancer TIA, Hruban RH, Theise ND. WHO Classification of Tumors of the Digestive System. International Agency for Research on Cancer; 2010.

2. Li H, Durbin R. Fast and accurate short read alignment with Burrows-Wheeler transform. Bioinformatics. 2009/05/20 ed. 2009;25(14):1754–60.

3. McKenna A, Hanna M, Banks E, Sivachenko A, Cibulskis K, Kernytsky A, et al. The Genome Analysis Toolkit: a MapReduce framework for analyzing next-generation DNA sequencing data. Genome Res. 2010/07/21 ed. 2010 Sep;20(9):1297–303.

4. Koboldt DC, Zhang Q, Larson DE, Shen D, McLellan MD, Lin L, et al. VarScan 2: Somatic mutation and copy number alteration discovery in cancer by exome sequencing. Genome Res. 2012 Feb 2;

5. Cingolani P, Platts A, Wang LL, Coon M, Nguyen T, Wang L, et al. A program for annotating and predicting the effects of single nucleotide polymorphisms, SnpEff: SNPs in the genome of Drosophila melanogaster strain w1118; iso-2; iso-3. Fly (Austin). 6(2):80–92.

6. Liu X, Jian X, Boerwinkle E. dbNSFP: a lightweight database of human nonsynonymous SNPs and their functional predictions. Hum Mutat. 2011 Aug;32(8):894–9.

7. Yost SE, Alakus H, Matsui H, Schwab RB, Jepsen K, Frazer KA, et al. Mutascope: sensitive detection of somatic mutations from deep amplicon sequencing. Bioinformatics. 2013 May 27;

8. Danecek P, Auton A, Abecasis G, Albers CA, Banks E, Depristo MA, et al. The Variant Call Format and VCFtools. Bioinformatics. 2011 Jun 7;27(15):2156–8.

9. San Lucas FA, Wang G, Scheet P, Peng B. Integrated annotation and analysis of genetic variants from next-generation sequencing studies with variant tools . Bioinforma . 2012 Feb 1;28 (3 ):421–2.
